# Correction to: The origins and molecular evolution of SARS-CoV-2 lineage B.1.1.7 in the UK

**DOI:** 10.1093/ve/veac119

**Published:** 2022-12-30

In the originally published version, the text ‘25th January, 3 May 2020,’ was duplicated in the first paragraph of the Results section. The duplicated text was redundant and has subsequently been removed.

